# Evaluation of the Relationship Between Suicide Behavior and SIRT-1 Gene in Patients with Schizophrenia and Other Psychotic Disorders

**DOI:** 10.1192/j.eurpsy.2022.909

**Published:** 2022-09-01

**Authors:** E. Uçak, M. Aydin, N. Koçak, K. Altınbaş

**Affiliations:** 1 Selçuk University, Faculty of Medicine, Psychiatry, Konya, Turkey; 2 Selçuk University, Faculty of Medicine, Genetics, Konya, Turkey

**Keywords:** schizophrénia, Genetics, Suicide, sirtuin1

## Abstract

**Introduction:**

Schizophrenia is a mental disorder with a high risk of suicide, which is one of the leading causes of early death in schizophrenia patients.

**Objectives:**

It was aimed to examine the relationship between the SIRT1 gene and suicidal behavior in patients with schizophrenia, to identify specific polymorphisms and to provide individual protective approaches by predicting suicidal behavior.

**Methods:**

100 patients with schizophrenia were included in our study. The SIRT1 gene was analyzed using the whole exome sequencing method, and 22 SNPs were identified. In addition, participants’ socio-demographic, psychiatric history, and suicidal behavior evaluation form data were recorded. A comparison was made between the two groups according to suicidal behavior.

**Results:**

When sociodemographic and psychiatric history of the participants were compared in terms of suicidal behavior, no significant difference was found. SIRT1 gene SNP; rs2236318; (TT genotype), rs10997870 (GG genotype) was associated about 4 times increased risk in suicidal behavior; rs41299232 (CC genotype) 3.7 times; rs7896005 (AA genotype) with 3.4 times also. Although rs201230502 (TC genotype) and rs36107781 (TC genotype) were more common in the group with suicidal behavior, they lost their significance in regression analysis due to the low number of cases.

**Conclusions:**

Our study showed that schizophrenia has many risks that increase suicidal behavior , but clinical and sociodemographic data are insufficient to predict suicidal behavior. Considering the inheritability of the disease and the effect of genetics on behavior, SIRT1 gene SNP; (rs2236318, rs10997870, rs41299232, rs7896005, rs201230502 and rs36107781) genotypes were found to be associated with suicidal behavior in schizophrenia patients.

**Disclosure:**

No significant relationships.

